# Conducting Randomized Controlled Trials for the Treatment of Enteric Fever

**DOI:** 10.1093/cid/ciu636

**Published:** 2014-08-05

**Authors:** Poojan Shrestha, Amit Arjyal

**Affiliations:** Patan Academy of Health Sciences, Oxford University Clinical Research Unit, Lalitpur, Nepal

To the Editor—We read with great interest the recent article by Meltzer et al, “A Large Outbreak of *Salmonella* Paratyphi A Infection Among Israeli Travelers to Nepal” [1], and we would like to raise a few issues. The authors made the point that randomized controlled trials (RCTs) are difficult to conduct in developing countries. However, in Nepal, over the last decade we have carried out a series of RCTs for the treatment of uncomplicated enteric fever [2–4]. Despite being constrained by limited infrastructure and research funding, we have made this a possibility by employing a novel design and method of carrying out RCTs for enteric fever, where we have formed a team of community health auxiliaries to visit patients at their homes where the treatments are administered and follow-up parameters measured. We have systematically evaluated several treatment options for the treatment of enteric fever in both adults and children, including an ongoing trial using intravenous medication. In fact, the trial currently under way, due to conclude soon, has ceftriaxone as one of its treatment arms. Such trials could also be adapted and carried out in other settings where the disease is endemic and would avert the difficulties of carrying out trials that the authors profess. Given the rapid emergence of resistance to commonly used antimicrobials against enteric fever, it is important to consistently carry out RCTs to determine the most optimum therapy, that which employs the shortest and simplest regimens to effectively cure the disease, and making use of novel methods might be the best way to do so in a setting such as ours beset by this neglected disease.

As difficult as it is to conduct such trials to the level of accepted standards, we would also like to point out that comparisons or documentation of treatment regimens, although not necessarily based on a randomized study, can also be immensely useful and practicable for quickly discerning and reporting failing treatments. However, in this study the treatment recommendations are based on the effects of the drugs on only 1 strain with a singular susceptibility pattern, whereas it must be borne in mind that in any endemic setting there are a multitude of strains and susceptibility patterns in play [5] that would have differing resistance to both ceftriaxone and azithromycin. Also, the effect of azithromycin seen may be a solitary effect as shown in other studies [6, 7] and not a synergistic one with ceftriaxone. Therefore, basing treatment regimens solely on comparisons presented in this study may not be appropriate.

It is also difficult to accurately diagnose enteric fever, and blood culture is positive in only 40%–60% of presumptive cases [8]. There indeed must have been other travelers returning from Nepal who had similar clinical features and needed the same courses of antimicrobials but whose blood cultures did not grow any organism. The proper identification of the subset of culture-negative patients is also immensely important. Given the good surveillance system in Israel, the report would have been more informative if the authors had included these patients as well.
